# Underwater Acoustic Target Recognition Based on Supervised Feature-Separation Algorithm

**DOI:** 10.3390/s18124318

**Published:** 2018-12-07

**Authors:** Xiaoquan Ke, Fei Yuan, En Cheng

**Affiliations:** Key Laboratory of Underwater Acoustic Communication and Marine Information Technology (Xiamen University) Ministry of Education, Xiamen University, Xiamen 361005, China; 23320171153169@stu.xmu.edu.cn (X.K.); chengen@xmu.edu.cn (E.C.)

**Keywords:** deep learning, autoencoder-decoder, Resonance-based Sparsity Signal Decomposition, target recognition, ship-radiated noise, feature-extraction, feature-separation

## Abstract

For the purpose of improving the accuracy of underwater acoustic target recognition with only a small number of labeled data, we proposed a novel recognition method, including 4 steps: pre-processing, pre-training, fine-tuning and recognition. The 4 steps can be explained as follows: (1) Pre-processing with Resonance-based Sparsity Signal Decomposition (RSSD): RSSD was firstly utilized to extract high-resonance components from ship-radiated noise. The high-resonance components contain the major information for target recognition. (2) Pre-training with unsupervised feature-extraction: we proposed a one-dimensional convolution autoencoder-decoder model and then we pre-trained the model to extract features from the high-resonance components. (3) Fine-tuning with supervised feature-separation: a supervised feature-separation algorithm was proposed to fine-tune the model and separate the extracted features. (4) Recognition: classifiers were trained to recognize the separated features and complete the recognition mission. The unsupervised pre-training autoencoder-decoder can make good use of a large number of unlabeled data, so that only a small number of labeled data are required in the following supervised fine-tuning and recognition, which is quite effective when it is difficult to collect enough labeled data. The recognition experiments were all conducted on ship-radiated noise data recorded using a sensory hydrophone. By combining the 4 steps above, the proposed recognition method can achieve recognition accuracy of 93.28%, which sufficiently surpasses other traditional state-of-art feature-extraction methods.

## 1. Introduction

When a ship moves in the water, it produces noise, called ship-radiated noise. Due to the unique characteristics of the radiated noise of different classes of ships, it is possible to identify a specific class of ships or even a specific ship by analyzing the ship-radiated noise. Recognition for ship-radiated underwater noise is one of the most important and challenging subjects in underwater acoustic signal processing. Traditionally, underwater acoustic target recognition depends on the decisions of well-trained sonar men, which can be highly inaccurate due to the need of continuous monitoring, and at times much affected by weather conditions. Hence, it is necessary to develop an automatic and robust recognizing system to replace humans work. Underwater acoustic target recognition is a complex pattern recognition problem. Due to the difficulty in collecting a large number of ship-radiated noise data, target recognition from ship-radiated noise is typically done under limited samples or even small samples.

Ship-radiated noise is composed of four generated sources: propulsion noise, propeller noise, auxiliary noise and hydrodynamic noise [1]. Quasi-periodic harmonics with low-frequency narrow-band components are produced by propulsion engines and propellers, whose amplitudes and frequencies are independent of ship speed [2]. Therefore, the harmonic elements play an important role in detection and recognition of underwater acoustic targets [3]. Due to generating mechanism of ship-radiated noise and effect of underwater acoustic channels, ship-radiated noise has the characteristics of oscillation, non-stationary and non-linearity [4]. Knowing that the harmonic elements or oscillatory components of ship-radiated noise play an important role in the detection and recognition of underwater acoustic targets, and motivated by the *oscillatory nature*, resonance-based sparsity signal decomposition (RSSD) [5] was proposed to extract the oscillatory signatures and condense noise. In pro-processing, RSSD is used to extract the oscillatory components (the high-resonance components) from ship-radiated noise. Once the high-resonance components are extracted, they can be used for further recognition of underwater acoustic targets.

To improve the recognition accuracy, many efforts were made to solve this intractable problem. Early work attempted to extract hand-crafted features from ship-radiated noise and feed them into different kinds of classifiers. Jian et al. in [6] extracted line spectrum features from ship-radiated noise and used support vector machine (SVM) to identify these features. More recently, Wei et al. in [7] introduced an approach for extracting ship-radiated noise based on $1_{\frac{1}{2}}$D spectrum and Principal Component Analysis (PCA) method. Zhang et al. in [8] extracted Mel Frequency Cepstrum Coefficients (MFCC), first-order differential MFCC, and second-order differential MFCC features and considered these features as the most effective traditional features for underwater acoustic target recognition. Meng and Yang in [9] designed a fusion feature of zero-crossing wavelength, peek-to-peek amplitude, and zero-crossing-wavelength difference for the recognition of underwater acoustic targets. Though the traditional hand-crafted features have contributed a lot to underwater acoustic target recognition, the process of extracting these features needs complicated engineering skill, domain expertise and prior knowledge. Especially the prior knowledge of ship-radiated noise, is usually difficult to be fully obtained because of the complicated ambient environment in oceans.

To solve the feature-extraction problems, many researchers also use neural networks to extract features. Utilizing neural networks to extract features requires less engineering skill, domain expertise and prior knowledge, but utilizing neural networks can also achieve a competitive performance even an outstanding performance compared with the traditional methods that extract the hand-crafted features. The performance of target recognition mainly relies on the distinction of the features extracted by neural networks. In [10], sparse Autoencoder (AE) was utilized to learn invariant features from spectral data of underwater targets. Then Cao et al. in [10] used Stacked Autoencoder (SAE) to extract high-level features and achieved a convincing recognition result. Some researchers attempted to introduce effective methods to modify the structure of neural networks. In [11], Yang et al. introduced competitive learning to Deep Belief Nets (DBN) and then built a so called competitive Deep Belief Nets (cDBN) for underwater acoustic target recognition. The idea of modifying the structure of a neural network always leads to surprising results.

**Figure 1:**
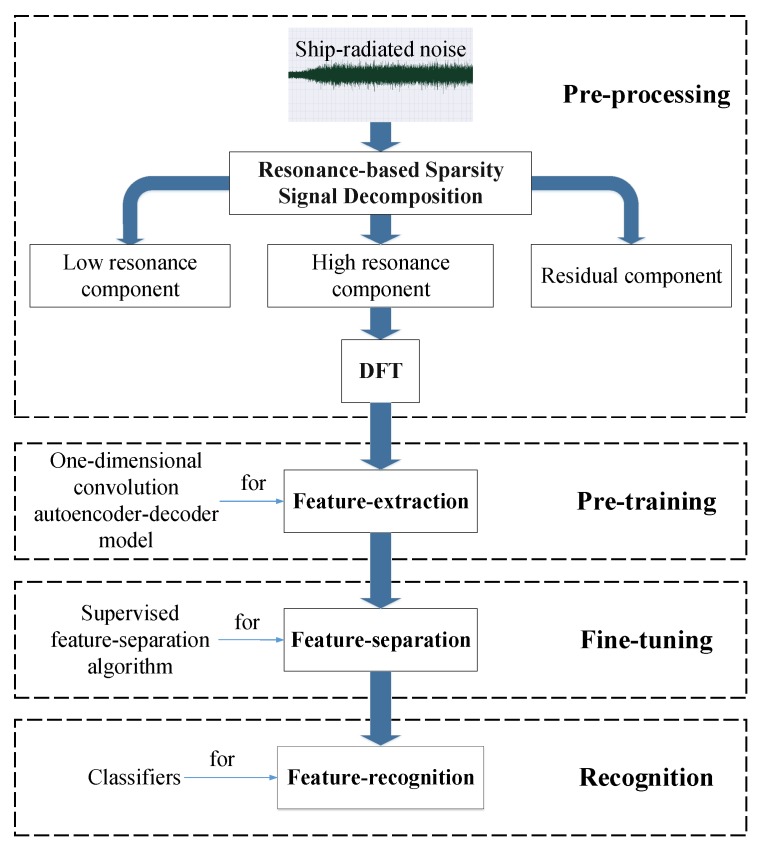
The whole process of underwater acoustic target recognition.

Underwater acoustic target recognition based on ship-radiated noise belongs to the small-sample-size recognition problem. As described in [11], unsupervised pre-training models (such as DBN) can make use of unlabeled data, and only a small number of labeled data are required. Knowing that an autoencoder-decoder is a type of artificial neural network used to learn efficient data coding in an *unsupervised* manner, we attempted to build an autoencoder-decoder model to fully make use of unlabeled data just like the DBN. Therefore, we firstly proposed a autoencoder-decoder model pre-trained by a large number of unlabeled data to fully learn the underlying laws from ship-radiated noise. Once the model is fully pre-trained by a large number of unlabeled data, the model can learn to extract features from ship-radiated noise as the unlabeled data always contain the general features of the interested underwater acoustic targets. In this paper, we call the phase of unsupervised pre-training *feature-extraction*. Besides this, we managed to use autoencoder-decoder to build a symmetrical model with encoding part of one-dimensional convolution and with decoding part of one-dimensional deconvolution. The autoencoder-decoders can be stacked to build a deeper model. Ranzato et al. in [12] proposed an unsupervised energy-based algorithm for separately pre-training layers of a Convolution Neural Network (CNN). Inspired by Ranzato’s work, we also applied an unsupervised *layer-wise* pre-training method to pre-train the model. Though the phase of unsupervised pre-training can extract features from ship-radiated noise, these features have not been identified with specific classes of ships. Then what we need to do is to further identify or separate these features with supervised training or fine-tuning. Inspired by SVM, we proposed a supervised *feature-separation* algorithm to fine-tune the model and separate the features extracted in the unsupervised pre-training. Finally, classifiers were trained to recognize the separated features and complete the recognition mission. The whole process of underwater acoustic target recognition is depicted in Figure 1.

The original and main contributions of this paper can be briefly summarized as follows: (1) we proposed a novel recognition method of 4 steps for underwater acoustic target recognition; (2) we carefully considered the oscillatory nature of ship-radiated noise and we proved the effectiveness of RSSD used in extracting “invariant” part of the ship-radiated noise for recognition; (3) we specially designed a totally new model with specific structures for extracting informative and invariant features for underwater acoustic target recognition; (4) we created a totally new universal loss function which named “feature-separation” algorithm for recognition.

The rest of this paper is organized as follows: The next section (Section 2) introduces RSSD for pre-processing. Section 3 describes the one-dimensional convolution autoencoder-decoder model for unsupervised pre-training. Section 4 fully explains the supervised feature-separation algorithm for supervised fine-tuning. Section 5 contains experiments and discussions. The last section (Section 6) is conclusion.

## 2. Resonance-Based Sparsity Signal Decomposition for Pre-Processing

The RSSD aims to decompose an objected signal into high-resonance, low-resonance and residual component, where the high-resonance component is a signal consisting of multiple simultaneous sustained oscillations, the low-resonance component is a signal consisting of non-oscillatory transients, and the residual component is Gaussian white noise [5]. The low-resonance component mostly contains the non-oscillatory transients, usually refer to pulse or transient interference, which is inevitably caused by complicated ocean environment during long-range transmission of the ship-radiated noise. On the contrary, the high-resonance component consists of the sustained oscillations, mostly composed of harmonic elements, which contain the main features for ship classification. Therefore, we abandon the low-resonance components and we use the high-resonance components for underwater acoustic target recognition. The RSSD algorithm for pro-processing mainly includes two parts: Tunable Q-Factor Wavelet Transform (TQWT) [13] and Morphological Component Analysis (MCA) [14].

The TQWT is a flexible fully-discrete wavelet transform which can be tuned according to the oscillatory behavior of the signal to which it is applied. The transform is based on real-valued scaling factors (dilation-factors) and is implemented using a perfect reconstruction over-sampled filter bank with real-valued sampling factors. The main parameters for the TQWT are Q-factor, redundancy *r*, and the number of stages *J*. The Q-factor, denoted *Q*, affects the oscillatory behavior of the wavelet, which is defined as [4]:

(1) $$Q=\frac{2-\beta }{\beta }$$

where β is high-pass scaling factor. A signal with a *higher Q-factor* reveals a *higher oscillatory* intensity in time-domain and, at the same time, better frequency concentration, and vice versa.

The aim of MCA is to construct the optimal sparse representation of the high-resonance and the low-resonance component, then separate these two components. Consider a given ship-radiated noise signal *x* as the sum of an oscillatory signal $x_{1}$, a non-oscillatory signal $x_{2}$ and a Gaussian white noise *n*:

(2) $$x=x_{1}+x_{2}+n$$

The signal *x* is a measured signal, $x_{1}$ and $x_{2}$ are to be determined in such a way that $x_{1}$ consists mostly of sustained oscillations and $x_{2}$ consists mostly of non-oscillatory transients. As described in [13], such a decomposition is necessarily nonlinear in *x*, and it cannot be accomplished using frequency-based filtering. One approach is to model $x_{1}$ and $x_{2}$ as having sparse representations using *high Q-factor* and *low Q-factor* wavelet transform respectively. In this case, a sparse representations of the signal *x* using both high Q-factor and low Q-factor TQWT jointly, makes the separation of $x_{1}$ and $x_{2}$ feasible. To separate $x_{1}$ and $x_{2}$, the approach is to minimize the following cost function [15]:

(3) $$\underset{w_{1},w_{2}}{\arg\min}||x-\Phi_{1}w_{1}-\Phi_{2}w_{2}{\left|\right|}_{2}+\sum_{j=1}^{J_{1}+1}\lambda_{1,j}\left|\right|w_{1,j}{\left|\right|}_{1}+\sum_{j=1}^{J_{2}+1}\lambda_{2,j}\left|\right|w_{2,j}{\left|\right|}_{1}$$

where *w* denotes coefficients of the TQWT, ${\left||.|\right|}_{2}$ denotes L2 norm, ${\left||.|\right|}_{1}$ denotes L1 norm, and $\Phi_{1}$ and $\Phi_{2}$ represent the inverse TQWT having high and low Q-factor respectively. The regularization parameters $\lambda_{1}$ and $\lambda_{2}$ are chosen by the user according the power of the noise. $w_{1}$ and $w_{2}$ are obtained by using Split Augmented Lagrangian Shrinkage Algorithm (SALSA) [5] to minimize Equation (3). After $w_{1}$ and $w_{2}$ are obtained, we set:

(4) $$x_{1}={\mathrm{TQWT}}_{1}^{-1}\left(w_{1}\right),\;x_{2}={\mathrm{TQWT}}_{2}^{-1}\left(w_{2}\right)$$

and then we can get the high-resonance component $x_{1}$ and the low-resonance component $x_{2}$. The residual component will be $n=x-x_{1}-x_{2}$.

## 3. One-Dimensional Convolution Autoencoder-Decoder Model for Unsupervised Pre-Training

The unsupervised pre-training autoencoder-decoder model can make good use of a large number of unlabeled data, so that only a small number of labeled data are required in the following supervised fine-tuning and recognition. This is quite effective when it is difficult to collect enough labeled ship-radiated noise data. The model will be described in detail as follows.

### 3.1. Building Units

Many unsupervised feature-extraction methods are based on encoder-decoder architecture [12]. In underwater acoustic target recognition, we want to build an autoencoder-decoder model to code the input spectrum, in other words, we want to automatically represent the input spectrum with code vectors. As the code vectors are abstractive representations of the inputs, they can be used to support high-level missions such as classification and pattern recognition. The model introduces convolution from CNN, but different from normal CNN, the model replaces two-dimensional convolution of the CNN with one-dimensional convolution. We managed to use the autoencoder-decoder to build a symmetrical model with encoding part of one-dimensional convolution and with decoding part of one-dimensional deconvolution. In this paper, we call the autoencoder-decoders *building units*. Note that a building unit is equivalent to a kernel (kernel, depth, and channel are the same in this paper).

### 3.2. The First Layer of the Model

**Figure 2:**
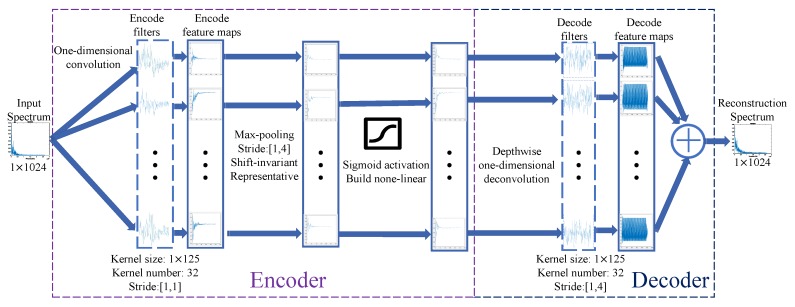
The first layer of the model.

Using the building units, we proposed a one-dimensional convolution autoencoder-decoder model to automatically code the input spectrum. The first layer of the model is depicted in Figure 2. A whole structure of the model will be described in next subsection. For simplicity, we denote all one-dimensional convolution as convolution and denote all one-dimensional deconvolution as deconvolution.

The first layer of the model contains two parts. The first part is an encoder. In Figure 2, size of the input spectrum is 1×1024, size of a convolution kernel is 1×125, and the number of kernels is 32. After the convolution, the number of feature maps will be 32, and because stride of the convolution is [1,1], size of each feature map will be 1×1024. A max-pooling layer and a sigmoid activation layer follow the convolution. The second part is a decoder, which reconstructs the input spectrum through depth-wise separable deconvolution [16]. Different from the traditional deconvolution, depth-wise separable deconvolution contains two steps: First, we apply deconvolution on a feature map. Size of the deconvolution is 1×125, which is corresponding to the convolution. Size of the feature map is 1×256, and stride of the deconvolution is [1,4], thus size of output of the deconvolution will be 1×1024, which is the same as that of the input spectrum. Second, we apply deconvolution on each of all 32 input feature maps, and thus we will get 32 outputs. Finally, reconstruction spectrum is the sum of the 32 outputs.

It is worth knowing that the proposed model has three specifically designed properties:

(1) Sparse and shift-invariant: Traditionally, sparsity is introduced by adding L1 regularization on feature maps, but in the model, max-pooling layers are introduced to achieve sparsity as well as shift-invariance. The sparsity can be viewed as limiting “capacity” of the networks, and it often results in more easily interpretable feature representations. The shift-invariance will make the model tolerate slight difference of the inputs. The reason for introducing the shift-invariance is to make the same class of input result in a same output. For the same class of ship-radiated noise, their spectrum usually only has slight difference, and shift-invariance will make outputs of the same class cluster together.
(2) Non-linear: Between the encoder and the decoder, there is a layer of sigmoid activation. This activation builds nonlinearity between two layers, which potentially makes the model build hierarchical representations of the inputs with the increasing of layers.
(3) Depth-wise separable: The model treats each depth independently. First, in unsupervised pre-training, features of each kernel are extracted separately and correlations between them are ignored. Then in supervised fine-tuning, the correlations between features of each kernel will be actually considered. The purpose of this operation is to make each feature more interpretable. This will be discussed in experiments section.

### 3.3. Stack Layers to Hierarchically Extract Features

In order to get more informative and invariant representations of the input spectrum, several layers of autoencoder-decoder are stacked to build a hierarchical model. We first introduce how we stacked the layers to build the model.

#### 3.3.1. Hyper-Parameter Optimization

The number of layers, kernel size of each layer, kernel number, kernel stride, type of activation function and so on are hyper-parameters of the model that need to be manually adjusted according to previous work, experiences or intuitions. The hyper-parameters of the model are listed in Table 1.

Since no previous work is available, we adjusted these hyper-parameters according to experiences and we conducted corresponding experiments to verify performance of the model of these adjustments. In other words, we explored each combination of the considered values in Table 1 (some combinations are not reasonable) and we conducted corresponding experiments to figure out whether these combinations or adjustments can improve performance (mainly refers to recognition accuracy) of the model. Through many experiments, the hyper-parameters that led to the best performance of the model were chosen, as listed in Table 1 the used values. The process of adjusting the hyper-parameters is similar to parameter optimization in [17] where conducted many experiments to choose the optimal parameters that led to the best performance of the network.

#### 3.3.2. The Structure of the Hierarchical Model

**Figure 3:**
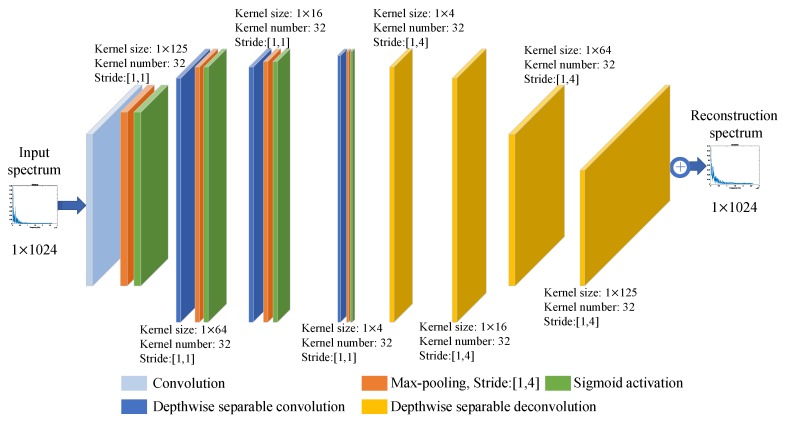
The structure of the hierarchical model.

The structure of the hierarchical model is depicted in Figure 3.

For layer 1, the encoder is a convolution with kernel size of 1×125, followed by a max-pooling layer and a sigmoid activation layer, and correspondingly, the decoder is a depth-wise separable deconvolution with kernel size of 1×125. The encoder of layer 2 is a depth-wise separable convolution with kernel size of 1×64, followed by a max-pooling layer and a sigmoid activation layer. The decoder of layer 2 is a depth-wise separable deconvolution with kernel size of 1×64. Layer 3 and layer 4 of the model are organized similarly to the previous two layers. depth-wise separable convolution and depth-wise separable deconvolution means that the model treats each kernel independently with convolution or deconvolution.

### 3.4. Learning Algorithm for Unsupervised Pre-Training

In Convolutional Networks [18], all the kernels are learned with a supervised gradient-based algorithm. However, a large number of labeled ship-radiated noise data are not always available to underwater target recognition. Due to the lack of labeled ship-radiated noise data, unsupervised pre-training with a large number of unlabeled data are introduced to initialize the network’s parameters. In other unsupervised pre-training models like DBN in [19], to fully make use of unlabeled data, they always use Contrastive Divergence (CD) algorithm to pre-train their models. It has been formally demonstrated that minimizing the CD is an approximation of maximizing likelihood [20]. Thus, weight connections of each layer of their models can have a good representative of the input data. However, their models do not have an encoder or decoder are forced to perform an expensive optimization in order to do inference even after learning the parameters. If the autoencoder-decoder is used, the general learning algorithm is only to minimize the reconstruction error between input spectrum and reconstruction spectrum. In other words, we search for feature maps that minimize the reconstruction error, while being not too different from outputs of the encoder. Note that the general learning algorithm is suitable for any encoder-decoder architecture, and not specific to a particular kind of features or architecture choices. In this paper, the learning algorithm for unsupervised pre-training is:

(5) $$\underset{w}{\arg\min}||X-\sum_{i=1}^{N}f_{i}*Z_{i}{\left|\right|}_{2}+{\lambda ||w||}_{2}$$

where *X* denotes the input spectrum of size 1×1024, $f_{i}\left(i=1,2,\cdots ,N\right)$ denotes deconvolution kernel, $Z_{i}$ is the *i*th feature map corresponding to the *i*th deconvolution kernel $f_{i}$, and ∗ denotes deconvolution. The second item in Equation (5) is L2 regularization used for avoiding over-fitting, *w* is the parameter of the model, and λ is “penalty” factor that controls the impact of L2 regularization on the learning algorithm. A larger value of λ will place more “penalty” on *w*. A proper value of λ can prevent *w* from getting too large while avoiding over-fitting. A typical value of λ is 0.0001. Similar to maximize the likelihood using CD, once the model is pre-trained by a large number of unlabeled ship-radiated noise data, the model can also fully learn the underlying laws from ship-radiated noise. Adaptive Moment Estimation (Adam) [21] is an effective way to minimize Equation (5).

**Figure 4:**
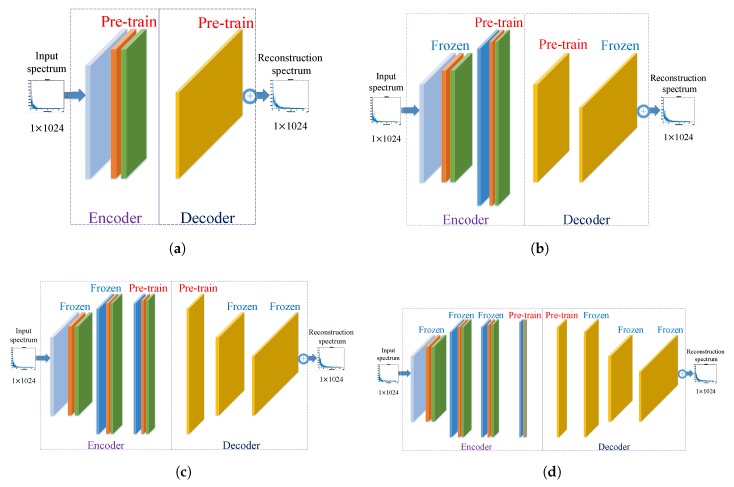
The procedure of layer-wise pre-training. (**a**) Pre-train layer1. (**b**) Pre-train layer 2. (**c**) Pre-train layer 3. (**d**) Pre-train layer 4.

Similar to separately pre-train layers of a network in [12], we pre-train the model layer-by-layer. The whole procedure of layer-wise pre-training is showed in Figure 4. Once layer 1 is pre-trained, all parameters of this layer will be frozen. Similarly, all parameters of layer 2 will be frozen if pre-training of this layer is finished. Layer 3 and Layer 4 are pre-trained in the same way as the previous two layers. Proposed by Hinton et al. in [22], layer-wise pre-training is a very effective method to handle the training issue of a deep network with a large number of parameters. Layer-wise pre-training can effectively avoid steeping into a local optimal and speed up the convergence of the following supervised fine-tuning.

## 4. Supervised Fine-Tuning and Feature-Separation

The model under unsupervised pre-training can extract informative features that support underwater acoustic target recognition. However, these features have not been identified with specific classes of ships. Then what we need to do is to further identify these features with supervised fine-tuning. As mentioned in [11], applying layer-wise pre-training and supervised fine-tuning, a deep neural network is built to obtain specific features from underwater acoustic targets. Inspired by this, we also apply fine-tuning to obtain more specific features from the targets. In this section, we first introduce a traditional supervised training algorithm for fine-tuning and then fully explain the supervised feature-separation algorithm for fine-tuning.

### 4.1. The Traditional Supervised Training Algorithm for Fine-Tuning

**Figure 5:**
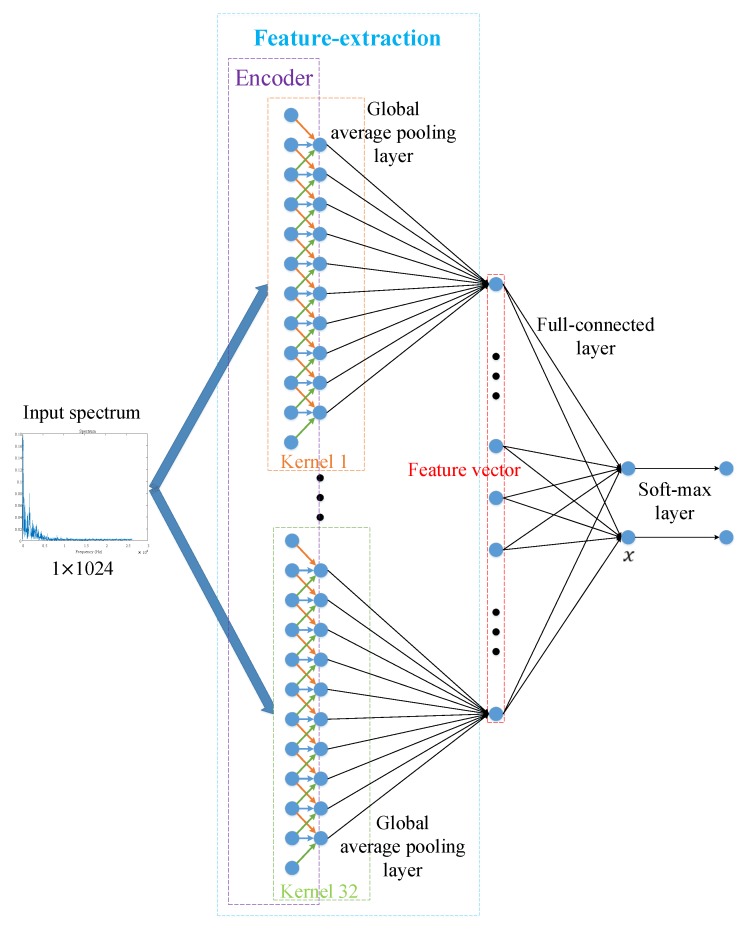
Procedure of the traditional supervised training algorithm for fine-tuning.

During unsupervised pre-training, feature maps are extracted from the encoder. The feature maps are abstractive representations of the inputs and that can be used for supporting target recognition. Further, these feature maps are fed into a global average pooling layer and *feature vectors* (as shown in Figure 5, 32 feature maps passing through a global average layer will become a feature vector of 32 dimensions) are obtained for fine-tuning. The traditional supervised training algorithm for fine-tuning is showed in Figure 5. In this algorithm, a fully-connected layer and a soft-max layer are used to fine-tune layer 1 of the model. The process of fine-tuning higher layers is the same as the fine-tuning of layer 1.

Assume that a feature vector (32 dimensions) passing through a fully-connected layer will become a vector $x=(x_{1},x_{2})$. The vector *x* passing through a soft-max layer will output [10]:

(6) $$\mathrm{softmax}{\left(x\right)}_{i}=\frac{e^{x_{i}}}{\sum_{i=1}^{2}e^{x_{i}}},i=1,2$$

Equation (6) means that the soft-max layer will turn a vector into a probability distribution in which each element corresponds to a specific class (e.g., a class of ships). Then in fine-tuning, we minimize cross-entropy between this probability distribution and the class label distribution $y=(y_{1},y_{2})$:

(7) $$H\left(y_{i},\frac{e^{x_{i}}}{\sum_{i=1}^{2}e^{x_{i}}}\right)=-\sum_{i=1}^{2}y_{i}\log\left(\frac{e^{x_{i}}}{\sum_{i=1}^{2}e^{x_{i}}}\right)$$

If we finish minimizing Equation (7), the probability distribution will become more closer to the class label distribution.

In fine-tuning, we can use Adam to minimize Equation (7). The traditional supervised training algorithm for fine-tuning can be summarized as follows:

(8) $$\underset{x}{\arg\min}\;H\left(y_{i},\frac{e^{x_{i}}}{\sum_{i=1}^{2}e^{x_{i}}}\right)=\underset{x}{\arg\min}\left[-\sum_{i=1}^{2}y_{i}\log\left(\frac{e^{x_{i}}}{\sum_{i=1}^{2}e^{x_{i}}}\right)\right]$$

### 4.2. The Supervised Feature-Separation Algorithm for Fine-Tuning

#### 4.2.1. Procedure of the Supervised Feature-Separation Algorithm

**Figure 6:**
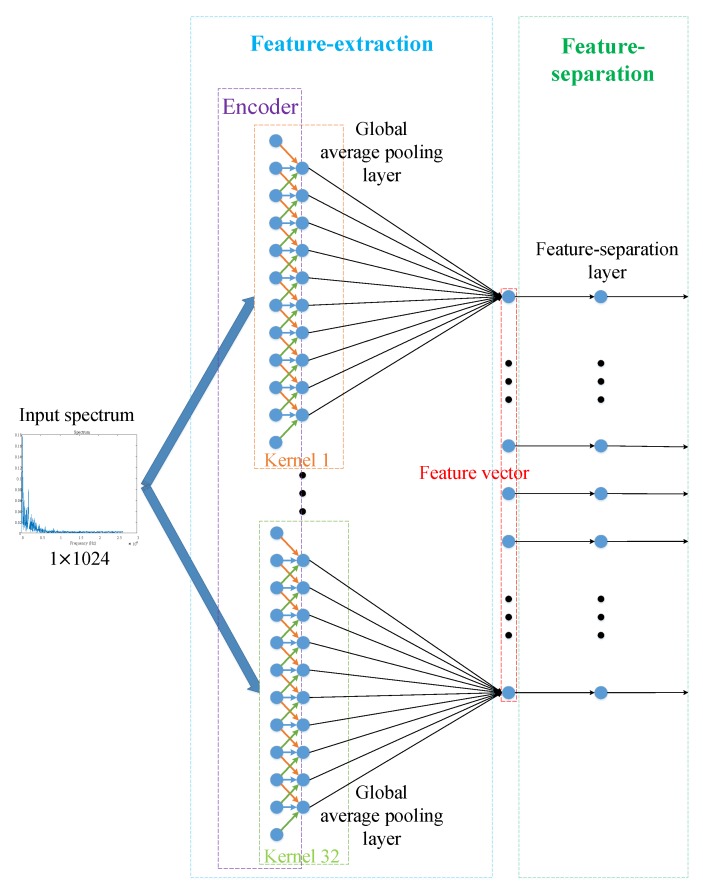
Procedure of the supervised feature-separation algorithm for fine-tuning.

Procedure of the supervised feature-separation algorithm is showed in Figure 6. In Figure 6, the feature-separation layer corresponds to the supervised feature-separation algorithm.

#### 4.2.2. Explanation of the Supervised Feature-Separation Algorithm

As features extracted by the model under unsupervised pre-training belong to different classes of ships, we aim to separate or identify these features according to different ship labels. In this algorithm, we use a hyperplane to separate these features.

Assume that in a 32-dimensional space, there is a hyperplane that can separate two classes of points, which means that one class of points lie on one side of the hyperplane, and the other class of points lie on the other side of the hyperplane. Then, our goal is to design the hyperplane that can handle the separation job. In a high-dimensional space, function of a hyperplane is:

(9) $$w^{T}x+b=0$$

where *T* denotes vector transpose, *x* denotes points on the hyperplane, *w* and *b* denote parameters of the hyperplane. In a 32-dimensional space, *w* is a vector of 32 dimensions, and *b* is a constant. Once the parameters (*w* and *b*) are fixed, then a unique hyperplane will be fixed, which means that a unique hyperplane corresponds to a unique set of parameters (*w* and *b*). For points on the hyperplane, Equation (9) will be satisfied, which means that if points are on the hyperplane, their distance to the hyperplane will be 0. When a hyperplane is fixed, we can use:

(10) $$f\left(x\right)=\left|w^{T}x+b\right|$$

to measure the distance f(x) from a random point *x* in the space to the hyperplane. By observing whether the sign of $w^{T}x+b$ is consistent with the sign of class label, it can be judged whether the classification is correct. Therefore, the positive and negative of $w^{T}x+b$ can be used to determine or indicate the correctness of the classification. More specifically, we use functional margin $\hat{\gamma }$ to denote the correctness of the classification:

(11) $$\hat{\gamma }=y\left(w^{T}x+b\right)$$

where *y* denotes class labels (−1 or 1). However, if we change *w* and *b* proportionally (such as changing them to 2w and 2b), value of the functional margin $\hat{\gamma }$ becomes twice the original value, while the hyperplane has not changed. Therefore, geometrical margin $\tilde{\gamma }$ is used to truly define the distance from points to the hyperplane:

(12) $$\tilde{\gamma }=\frac{\hat{\gamma }}{{\left||w|\right|}_{2}}=\frac{y(w^{T}x+b)}{{\left||w|\right|}_{2}}$$

**Figure 7:**
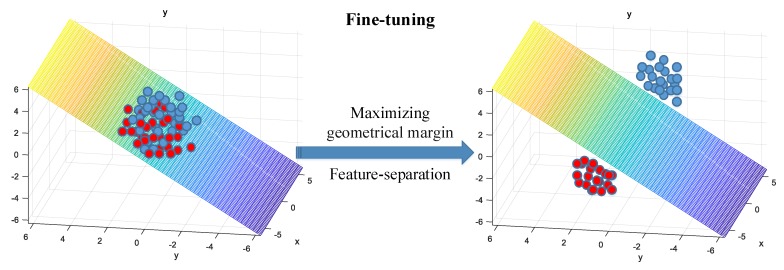
The process of feature-separation.

As geometrical margin $\tilde{\gamma }$ measures the distance from points to the hyperplane, it can also be used to define the distance from high-dimensional feature vectors to the hyperplane. More specially, we view a high-dimensional feature vector as a point in the high-dimensional space, then we can use Equation (12) to calculate its distance to the hyperplane. Now, we are going to make feature vectors belong to one class of ships lie on one side of the hyperplane, and make feature vectors belong to the other class of ships lie on the other side of the hyperplane. The approach is to maximize Equation (12), so that different classes of feature vectors will both move away from the hyperplane, and that completes the separation of feature vectors of different classes. In conclusion, the supervised feature-separation algorithm can be explained as:

(13) $$\underset{w,b,x}{\arg\max}\frac{y(w^{T}x+b)}{{\left||w|\right|}_{2}}=\underset{w,b,x}{\arg\min}\frac{1}{\frac{y(w^{T}x+b)}{{\left||w|\right|}_{2}}}$$

where *x* denotes feature vectors, *y* denotes class labels (−1 or 1) corresponding to *x*, *w* and *b* are parameters of the hyperplane that can be trained automatically. In fine-tuning, we set parameters (*w* and *b*) of the hyperplane to be trainable, and meanwhile set the parameters of the model to be *trainable* too. The model is fine-tuned with a small number of labeled ship-radiated noise data. Along with the process of fine-tuning, feature vectors of different classes of ships will gradually move away from the hyperplane (see Figure 7), and the hyperplane will eventually be fixed. Similarly, we can also use Adam to minimize Equation (13).

Compared with the traditional supervised training algorithm (Figure 5), the supervised feature-separation algorithm (Figure 6) can reduce the number of parameters of a network. For example, in Figure 5, the fully-connected layer has 66 parameters (32×2 weights and 1×2 biases), while a feature-separation layer only has 33 parameters (the hyperplane has 33 parameters, *w* is a vector of 32 dimensions, and *b* is a constant), which means that the supervised feature-separation algorithm can reduce 50% of the parameters. More importantly, the supervised feature-separation algorithm provides a perfect interface connecting to different kinds of classifiers directly, which means that feature vectors passing through a feature-separation layer can be fed to classifiers directly without reducing their dimensions (As shown in Figure 6, before passing through the feature-separation layer, the feature vectors have 32 dimensions, and after passing through the feature-separation layer, the feature vectors still have 32 dimensions, while in Figure 5, feature vectors passing through a fully-connected layer only have 2 dimensions left). Besides this, the feature-separation algorithm with dimension-invariance also builds a bridge connecting unsupervised pre-training and supervised fine-tuning, which is quite suitable for target recognition of small samples. Last but not least, we created a universal loss function (Equation (13)) for fine-tuning, which makes the process of fine-tuning more explanatory and intuitive, because we make the meaning of the feature-separation layer quite clear–it is a *hyperplane*.

## 5. Experiments and Discussion

### 5.1. Experiment Dataset

**Figure 8:**
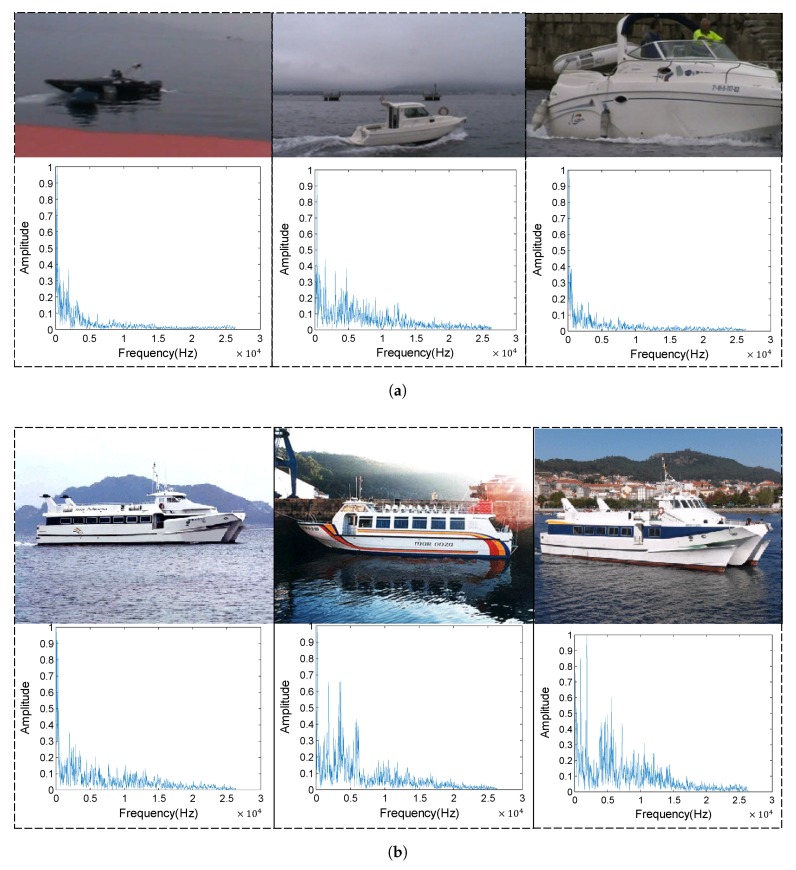
Some originally recorded samples. (**a**) Pictures and spectrum of the recorded samples in class 1. (**b**) Pictures and spectrum of the recorded samples in class 2.

All data of ship-radiated noise come from a database called ShipsEar [23]. During 2012 and 2013 the sounds of many different classes of ships were recorded on the Spanish Atlantic coast and were included in the ShipsEar database (available at http://atlanttic.uvigo.es/underwaternoise/). The recordings were made with autonomous acoustic digitalHyd SR-1 recorders, manufactured by MarSensing Lda (Faro, Portugal). This compact recorder includes a hydrophone with a nominal sensitivity of −193.5 dB re 1V/1 uPa and a flat response in the 1 Hz–28 kHz frequency range. We choose two classes of ship-radiated noise as labeled data, and rest of the ship-radiated noise will be treated as unlabeled data for unsupervised pre-training. The pre-training data also include underwater ambient noise. There are 9983 samples in class 1, and 22,523 in class 2. Each sample lasts approximately 0.03 s with sampling frequency of 52,734 Hz. We set 80% of labeled data for supervised fine-tuning, and we set remaining 20% of labeled data for recognition accuracy testing. We randomly picked up some originally recorded samples in class 1 and class 2 and we show them in Figure 8. In Figure 8, class 1 denotes motorboats and class 2 denotes passenger ferries.

### 5.2. Experiment of Pre-Processing

**Figure 9:**
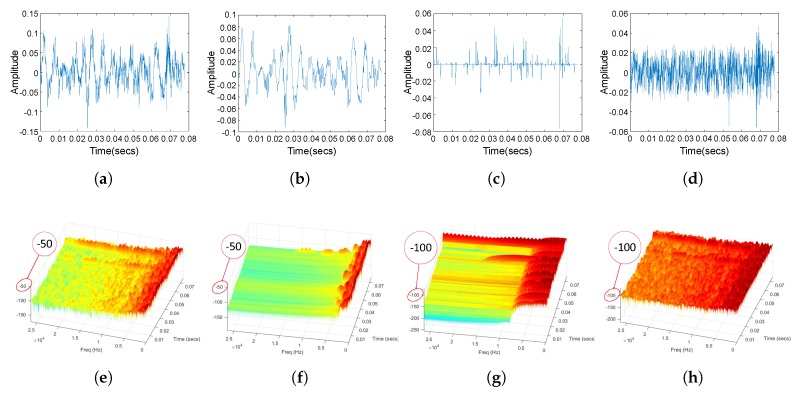
RSSD result. (**a**) Waveform of the originally recorded signal. (**b**) Waveform of the high-resonance component. (**c**) Waveform of the low-resonance component. (**d**) Waveform of the residual component. (**e**) 3-D spectrogram of the originally recorded signal. (**f**) 3-D spectrogram of the high-resonance component. (**g**) 3-D spectrogram of the low-resonance component. (**h**) 3-D spectrogram of the residual component.

In pre-processing, RSSD was utilized to extract the high-resonance components from ship-radiated noise. We set $Q_{1}=4$, $r_{1}=3$, and $J_{1}=32$ for the high-Q TQWT [4]. For the low-Q TQWT, we set $Q_{2}=1$, $r_{2}=3$, and $J_{2}=3$ [4]. We randomly picked up a originally recorded signal and demonstrated its RSSD result in Figure 9. RSSD decomposes the originally recorded signal into high-resonance, low-resonance and residual component, which are shown in Figure 9b–d, respectively, and the originally recorded signal is shown in Figure 9a. The spectrum of the original recorded signal, the high-resonance component, the low-resonance component and the residual component are shown in Figure 9e–h, respectively.

**Figure 10:**
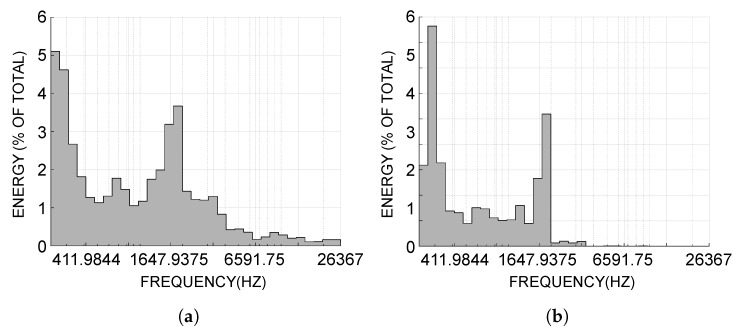
(**a**) Energy distribution of the original signal. (**b**) Energy distribution of the high-resonance component.

It is clear from Figure 9 that the high-resonance component contains much less noise and its spectrogram is much more pure compared with that of the original signal. Besides this, we can know that the high-resonance component contains the most part of energy of the original signal and its spectrogram is the most similar with that of the original signal. We further demonstrate the energy distribution of the original signal and the high-resonance component in Figure 10.

**Figure 11:**
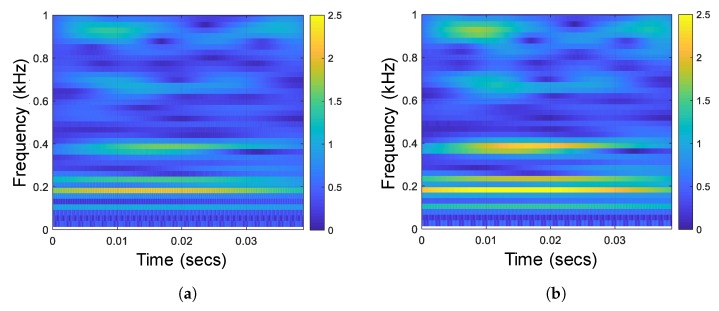
(**a**) Spectrogram of the original signal. (**b**) Spectrogram of the high-resonance component.

Figure 10b shows that energy of the high-resonance component mostly concentrates on a narrow band in low frequency. This will improve the recognition accuracy of underwater acoustic targets because the *harmonic elements*, especially in the low frequency are the main features (also known as *line spectrum*) of the ship-radiated noise [3]. A similar result is showed in Figure 11. It is obviously from Figure 11 that the harmonic elements of the high-resonance component in the low frequency are much more clear than that of the original signal.

For further evaluating the effectiveness of the RSSD used in pre-processing, we calculated Spectral Correlation Coefficient $C_{xy}$ [24] of the original signals and their high-resonance components, respectively. $C_{xy}$ is almost independent of the sailing speed of ships [24]. The same class of ships tend to have a large $C_{xy}$ while $C_{xy}$ between different classes of ships is much smaller [24]. The $C_{xy}$ is defined as follow:

(14) $$C_{xy}=\frac{\int_{f_{1}}^{f_{2}}N_{xx}\left(f\right)\cdot N_{yy}\left(f\right)\;df}{\sqrt{\int_{f_{1}}^{f_{2}}N_{xx}^{2}\left(f\right)\;df\cdot \int_{f_{1}}^{f_{2}}N_{yy}^{2}\left(f\right)\;df}}$$

where N(·) denotes spectrum. Table 2 is the results of calculating $C_{xy}$.

Table 2 shows that the high-resonance components tend to have a larger $C_{xy}$ between the same class of ships and a relatively smaller $C_{xy}$ between the different classes of ships compared with that of the original signals. Table 2 shows that after applying RSSD, the “invariant” part of the ship-radiated noise is extracted. This “invariant” part tends to make the same class of ships more related and to make the different classes of ships more independent. We can make a conclusion that RSSD can make the different classes of ships more easily distinguishable. The following experiments will be all conducted on the high-resonance components of the ship-radiated noise.

### 5.3. Experiment of Unsupervised Pre-Training

**Figure 12:**
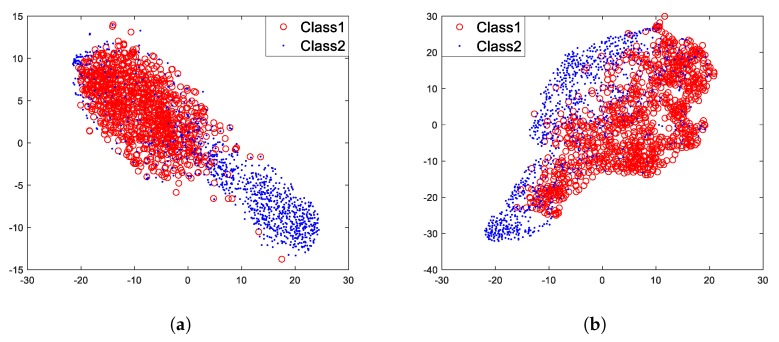
Feature visualization results. (**a**) MFCC features (23 dimensions). (**b**) Feature vectors (32 dimensions) of layer 1 under unsupervised pre-training.

To illustrate the performance of the model, T-Distribution Stochastic Neighbor Embedding (t-SNE) [25] was used to visualize the feature vectors. The visualization technique t-SNE can map high-dimensional feature vectors into 2 dimensions and show distributions of the high-dimensional feature vectors. For this moment, the model has not been fine-tuned with labeled data, and data used for visualization are not included in pre-training. Data used for visualization come from a small part of the labeled data. As mentioned in [11], traditional hand-crafted features used for underwater acoustic target recognition include Mel Frequency Cepstrum Coefficients (MFCC), waveform, auditory and wavelet features of the ship-radiated noise. However, in [11], experiments have proved that the MFCC features are the best traditional hand-crafted features for the recognition, so we only use MFCC features for comparison. After pre-training the model with a large number of unlabeled data, we can feed it with labeled data to extract feature vectors from each layer. What we obtained were feature vectors after using labeled data to do feed-forward inference, and we used t-SNE to visualize these feature vectors. For comparison, we also visualized MFCC features of the same labeled ship-radiated noise data. Figure 12a is the visualization result of MFCC features, while Figure 12b is the visualization result of feature vectors of layer 1 under unsupervised pre-training.

Figure 12a shows that two different classes of MFCC features mainly cluster into two parts, which indicates that to a certain extent, MFCC features are quite effective for target classification and recognition. In Figure 12b, the distributions of feature vectors of layer 1 is similar to the distributions of MFCC features. For this, we can preliminarily think that the model under unsupervised pre-training can learn potential laws from the ship-radiated noise.

**Figure 13:**
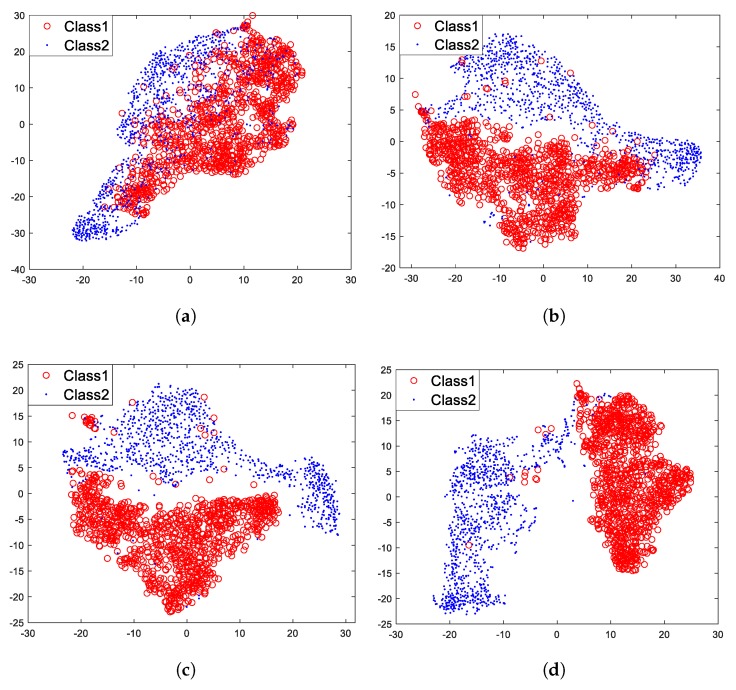
Feature visualization under unsupervised pre-training. (**a**) Layer 1. (**b**) Layer 2. (**c**) Layer 3. (**d**) Layer 4.

Then, we used t-SNE to visualize feature vectors of each layer of the model under unsupervised pre-training, which are showed in Figure 13.

Figure 13 shows that for layer 1, two different classes of feature vectors mix together, however, with the increasing of layers, two different classes of feature vectors gradually move away from each other and eventually cluster into two parts (Figure 13d). It is undoubted that two different classes of feature vectors of layer 4 are more distinguishable than that of layer 1. It is worth knowing that the three specifically designed properties (sparse and shift-invariant, non-linear, depth-wise separable) of the model mainly contribute to these results. The shift-invariance makes the same class of feature vectors cluster together, and the non-linearity makes the feature vectors gradually become more informative with the increasing of layers. For this we can draw a conclusion: with specifically designed properties, the proposed hierarchical model can gradually extract more informative and invariant features for target recognition with the increasing of layers.

### 5.4. Experiment of Supervised Fine-Tuning

**Figure 14:**
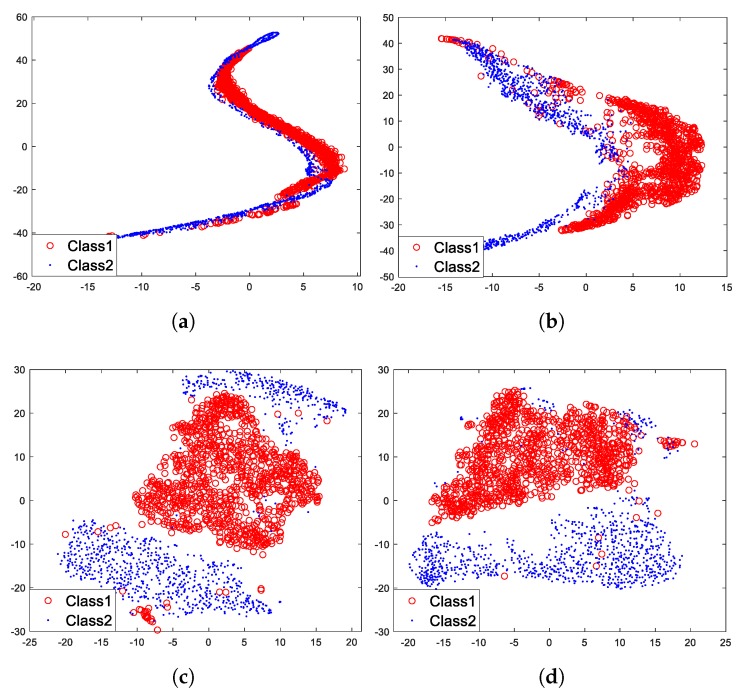
Feature visualization after fine-tuning with the traditional supervised training algorithm. (**a**) Layer 1. (**b**) Layer 2. (**c**) Layer 3. (**d**) Layer 4.

The model under unsupervised pre-training is capable of extracting informative and invariant features for target recognition. Then, we fine-tuned the model with the traditional supervised training algorithm (Figure 5), intending to figure out whether the algorithm can improve recognition performance. The process of this section is similar to Section 5.3, what is different is that the model has been fine-tuned with labeled data. Figure 13 has demonstrated the visualization results of feature vectors of each layer under unsupervised pre-training, and similar results are depicted in Figure 14 but after fine-tuning with the traditional supervised training algorithm.

Figure 14 shows that after fine-tuning with the traditional supervised training algorithm, two different classes of feature vectors of each layer tend to move away from each other compared with that of Figure 13. Two different classes of feature vectors of each layer become more distinguishable.

**Figure 15:**
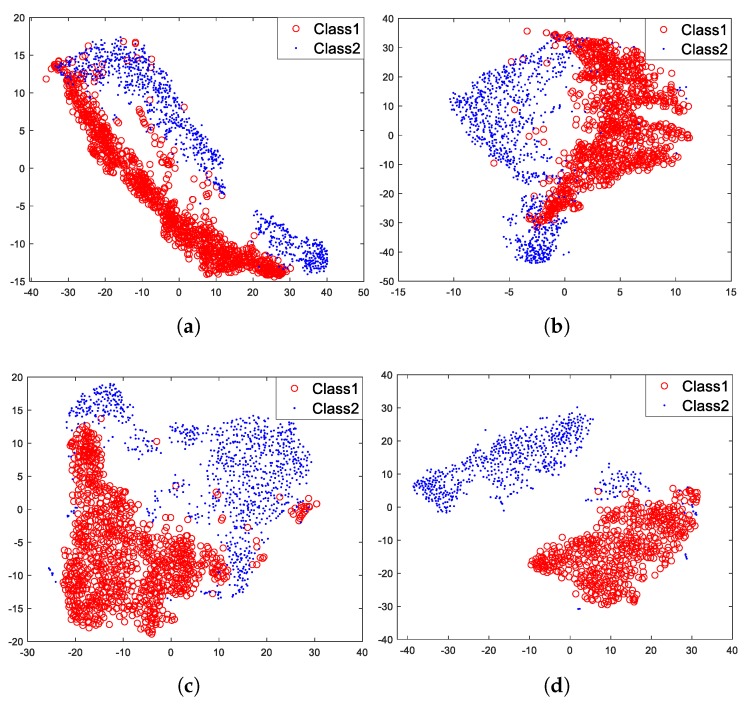
Feature visualization after fine-tuning with the supervised feature-separation algorithm. (**a**) Layer 1. (**b**) Layer 2. (**c**) Layer 3. (**d**) Layer 4.

Then we fine-tuned the model with the supervised feature-separation algorithm (Figure 6). For a fair comparison, all experimental conditions were the same. The visualization results are depicted in Figure 15.

Figure 15 shows that in comparison with Figure 13, two different classes of feature vectors of each layer also tend to move away from each other after fine-tuning with the supervised feature-separation algorithm. We can imagine that there is a hyperplane between the two classes of feature vectors that always tend to keep these two classes of feature vectors separated. Especially layer 1 (Figure 15a), two different classes of feature vectors are almost separated away from each other after fine-tuning with the supervised feature-separation algorithm, which verifies the effectiveness of the algorithm.

In the traditional supervised training algorithm, the fully-connected layer tends to map the “distributed feature representations” to different sample space, which means that the fully-connected layer focuses on each dimension of a feature vector and identify each dimension of a feature vector separately, while the feature-separation layer views a feature vector as an entirety (a point in the high-dimensional space) and directly separates it from others with a hyperplane. Since we apply depth-wise separable convolution and deconvolution in pre-training, the correlations between features of each kernel are ignored. However, when we apply the supervised feature-separation algorithm in fine-tuning, the correlations between features of each kernel will be actually considered because we view a feature vector as an entirety. The operation of “first splitting up and then combining together” makes each feature more interpretable because we both consider the independence and the correlations of features of each kernel but in a separated way. The distributions of two different classes of feature vectors are different passing through a fully-connected layer or a feature-separation layer, but they both become more distinguishable.

### 5.5. Experiment of Underwater Acoustic Target Recognition

This section we will discuss the performance of underwater acoustic target recognition of the model. As mentioned in [11], MFCC features have been experimentally proved to be the best hand-crafted features for the recognition, we only use MFCC features for comparison. We extracted MFCC features of training data to train SVM classifiers and extracted MFCC features of testing data to calculate recognition accuracy.

As shown in Figure 13, feature vectors extracted by the model under unsupervised pre-training can be used for target recognition, because the feature vectors are distinguishable with different classes just like the MFCC features. Therefore, we added labels to the feature vectors and used them for target recognition. More specifically, after pre-training the model with unlabeled data, we fed it with training data (with labels) and extracted feature vectors from each layer. Then the feature vectors (with labels) can be used to train SVM classifiers. For testing phase, we fed testing data to the model and extracted feature vectors to verify the recognition performance of the model.

**Figure 16:**
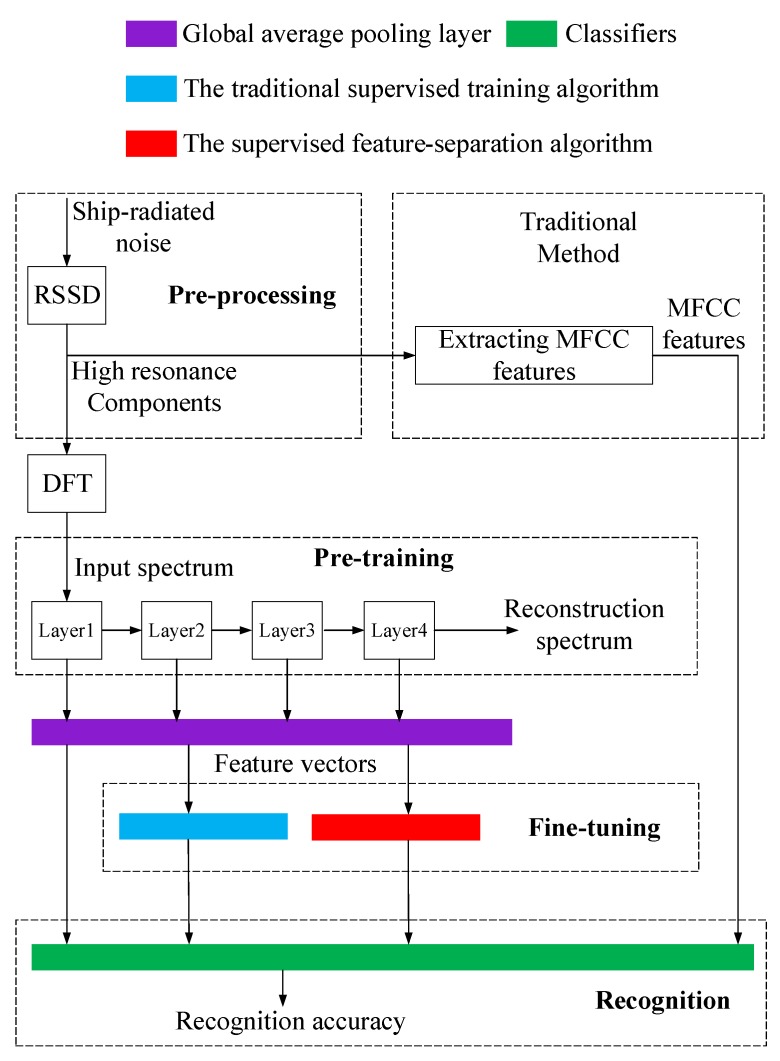
Procedure of the underwater acoustic target recognition experiment.

After evaluating the recognition performance of the model under pre-training, we further evaluated the recognition performance of the model after fine-tuning. We used both the traditional supervised training algorithm and the supervised feature-separation algorithm for fine-tuning. The whole experiment procedure of this section is depicted in Figure 16.

Parameters of the SVM classifiers were selected by using 10-fold cross-validation. In Table 3, average recognition accuracy over 10 random trials is reported.

**Figure 17:**
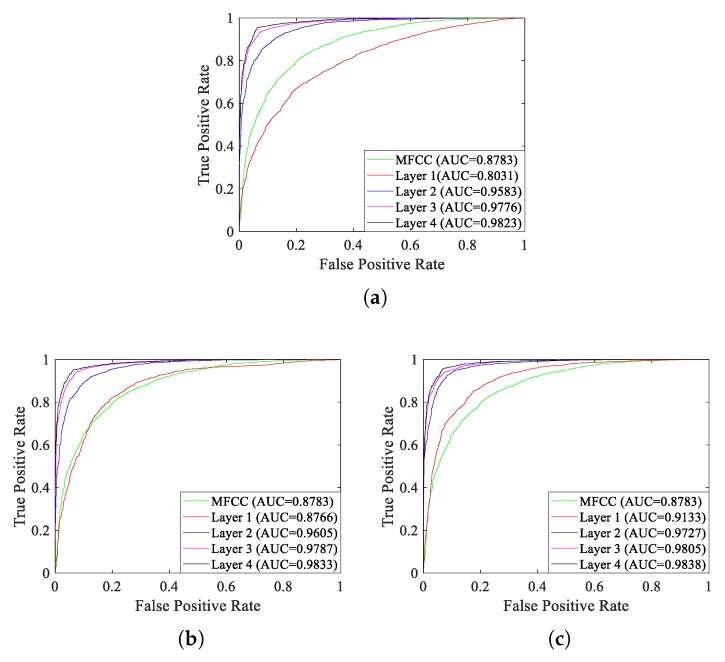
ROC curves of each layer of the model. (**a**) Unsupervised pre-training. (**b**) Fine-tuning with the traditional supervised training algorithm. (**c**) Fine-tuning with the supervised feature-separation algorithm.

As shown in Table 3, recognition accuracy of MFCC features surpasses the feature vectors of layer 1 of the model under pre-training, however, with the increasing of layers, recognition accuracy of the model will greatly improve, which indicates that the model can indeed extract informative and invariant features for target recognition. The recognition accuracy will improve after supervised fine-tuning, especially layer 1, which improves almost 10.5% of accuracy after fine-tuning with the supervised feature-separation algorithm. Besides this, no matter pre-training or fine-tuning, recognition accuracy improves with the increasing of layers. Generally speaking, after fine-tuning with the supervised feature-separation algorithm, layer 4 achieves the highest recognition accuracy of 93.28%. We draw ROC (Receiver Operating Characteristic) curves with AUC (Area Under Curve) values to further evaluate the recognition performance of the model, which are depicted in Figure 17. The one has the larger AUC value has the better recognition performance.

**Figure 18:**
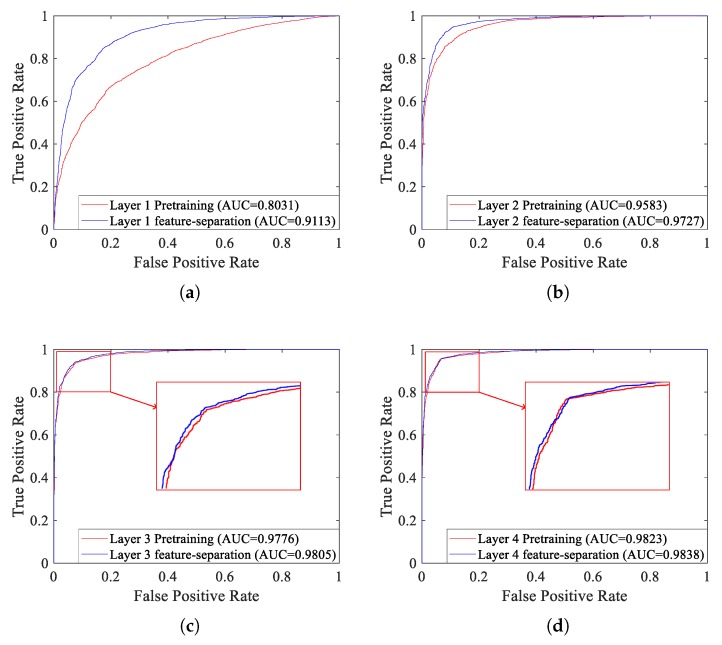
ROC curves of each layer under unsupervised pre-training and after fine-tuning with the supervised separation algorithm. (**a**) Layer 1. (**b**) Layer 2. (**c**) Layer 3. (**d**) Layer 4.

As shown in Figure 17, with the increasing of layers, the recognition performance will improve no matter unsupervised pre-training or supervised fine-tuning, just as mentioned above. In order to further verify the effectiveness of the supervised feature-separation algorithm, we draw ROC curves of each layer under unsupervised pre-training and after fine-tuning with the supervised separation algorithm, which are depicted in Figure 18.

As shown in Figure 18, compared with pre-training, no matter which layer, the recognition performance will improve after fine-tuning with the supervised feature-separation algorithm, just as mentioned above. Comparing Figure 18a and Figure 18d, we can know that recognition performance of layer 1 was greatly improved after fine-tuning with the supervised separation algorithm, while the recognition performance of layer 4 was improved only slightly. For this, we can draw a conclusion that the supervised feature-separation algorithm is capable of separating the mixed features (just like Figure 13a), rather than further separating the features that are already separated (just like Figure 13d).

**Figure 19:**
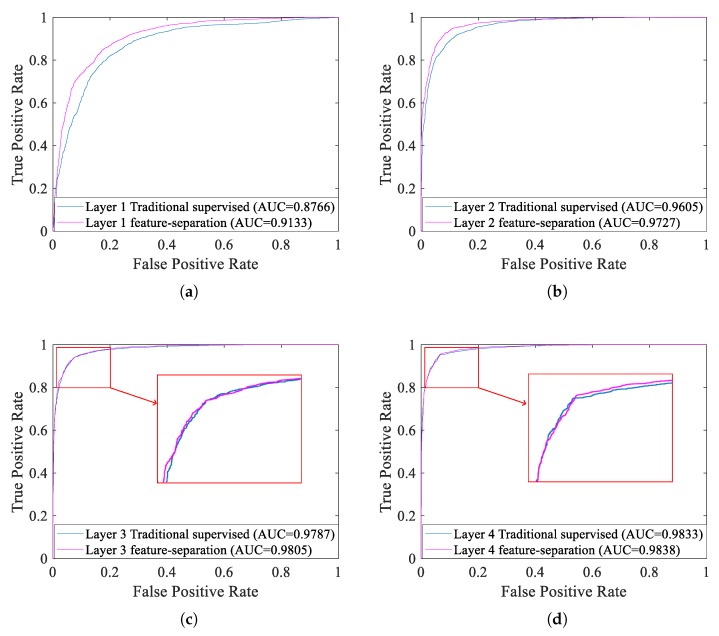
ROC curves of each layer after fine-tuning with the traditional supervised training algorithm and with the supervised feature-separation algorithm. (**a**) Layer 1. (**b**) Layer 2. (**c**) Layer 3. (**d**) Layer 4.

Lastly, we draw ROC curves of each layer after fine-tuning with the traditional supervised training algorithm and with the supervised feature-separation algorithm, which are showed in Figure 19.

From Figure 19a we can indicate that the recognition performance of the supervised feature-separation algorithm is better than the traditional supervised training algorithm when feature vectors mix together (just like Figure 13a). This experimental result conforms our expectation, because we indeed purposely use a hyperplane to separate the two different classes of feature vectors. For this, we can confirm the conclusion: the supervised feature-separation algorithm is quite capable of separating the mixed features (just like Figure 13a). However, with the increasing of layers, the advantage of the supervised feature-separation algorithm over the traditional supervised training algorithm become less outstanding. Just as shown in Figure 19c,d, when two different classes of feature vectors no longer mix together, ROC curves of the two supervised algorithms overlap together, which indicates that the both two supervised algorithms have the same recognition performance, and the advantage of the supervised feature-separation algorithm become less outstanding.

## 6. Conclusions

For the purpose of improving accuracy of underwater acoustic target recognition under the condition of small size of labeled data, we proposed a novel recognition method of 4 steps including pre-processing, pre-training, fine-tuning and recognition. In pre-processing, RSSD was utilized to extract the high-resonance components from ship-radiated noise. Experiments showed that after pre-processing with RSSD, different classes of ships become more easily distinguishable. In pre-training, we proposed the one-dimensional convolution autoencoder-decoder model to extract features from the high-resonance components and experiments showed that pre-trained with a large number of unlabeled data, the model can gradually extract more informative and invariant features for underwater acoustic target recognition with the increasing of layers. In fine-tuning, the supervised feature-separation algorithm was proposed to further separate the features extracted in pre-training and experiments showed that the algorithm is quite capable of separating the mixed features. By combining the 4 steps, the model can achieve recognition accuracy of 93.28%, which sufficiently surpasses other traditional state-of-art feature-extraction methods.

## Figures and Tables

**Table 1:** Hyper-parameters of the Model.

Hyper-Parameters		Considered Values	Used Values
Layer 1	Kernel size	1 × 256, 1 × 125, 1 × 64, 1 × 32, 1 × 16, 1 × 8, 1 × 4, 1 × 2	1 × 125
	Kernel number	128, 64, 32, 16, 4	32
	Kernel stride	[1,1], [1,2], [1,4]	[1,1]
Layer 2	Kernel size	1 × 64, 1 × 32, 1 × 16, 1 × 8, 1 × 4, 1 × 2	1 × 64
	Kernel number	128, 64, 32, 16, 4	32
	Kernel stride	[1,1], [1,2], [1,4]	[1,1]
Layer 3	Kernel size	1 × 32, 1 × 16, 1 × 8, 1 × 4, 1 × 2	1 × 16
	Kernel number	128, 64, 32, 16, 4	32
	Kernel stride	[1,1], [1,2], [1,4]	[1,1]
Layer 4	Kernel size	1 × 16, 1 × 8, 1 × 4, 1 × 2	1 × 4
	Kernel number	128, 64, 32, 16, 4	32
	Kernel stride	[1,1], [1,2], [1,4]	[1,1]
The number of layers		1, 2, 3, 4, 5	4
Type of activation function		Sigmoid, Tanh, ReLU, Leakey ReLU	Sigmoid
Max-pooling stride		[1,4], [1,8]	[1,4]
Depthwise separable		Yes, not	Yes

**Table 2:** The Results of Calculating $C_{xy}$.

The Original Signals			The High-Resonance Components		
$C_{xy}$	${\left(\mathrm{Class}\;1\right)}_{x}$	${\left(\mathrm{Class}\;2\right)}_{x}$	$C_{xy}$	${\left(\mathrm{Class}\;1\right)}_{x}$	${\left(\mathrm{Class}\;2\right)}_{x}$
${\left(\mathrm{Class}\;1\right)}_{y}$	0.5830	0.5389	${\left(\mathrm{Class}\;1\right)}_{y}$	0.6191	0.5496
${\left(\mathrm{Class}\;2\right)}_{y}$	0.5389	0.5409	${\left(\mathrm{Class}\;2\right)}_{y}$	0.5496	0.5622

**Table 3:** Average Recognition Accuracy.

Methods	Features	Dimensions	Accuracy/%
Traditional	MFCC	23	79.62
Unsupervised pre-training	Layer 1	32	72.98
	Layer 2	32	88.33
	Layer 3	32	92.29
	Layer 4	32	92.75
Fine-tuning with the traditional supervised training algorithm	Layer 1	32	80.75
	Layer 2	32	89.29
	Layer 3	32	92.49
	Layer 4	32	93.24
Fine-tuning with the supervised feature-separation algorithm	Layer 1	32	83.37
	Layer 2	32	91.51
	Layer 3	32	92.83
	Layer 4	32	*93.28*
